# Regulation of Oxidative Stress and Cardioprotection in Diabetes Mellitus

**DOI:** 10.2174/157340308786349426

**Published:** 2008-11

**Authors:** Tetsuya Hayashi, Tatsuhiko Mori, Chika Yamashita, Masatoshi Miyamura

**Affiliations:** 1 Department of Internal Medicine III, Osaka Medical College; 2 Clinical Trial Center, Osaka Medical College Hospital; 3 Laboratory of Pathological and Molecular Pharmacology, Osaka University of Pharmaceutical Sciences, Takatsuki, Osaka, Japan

**Keywords:** Oxidative stress, diabetes mellitus, sleep apnea, hypoxia, remodeling.

## Abstract

Analysis of the Framingham data has shown that the risk of heart failure is increased substantially among diabetic patients, while persons with the metabolic syndrome have an increased risk of both atherosclerosis and diabetes mellitus. Sleep apnea may be related to the metabolic syndrome and systemic inflammation through hypoxia, which might also cause the cardiac remodeling by increased oxidative stress. On the other hand, the renin-angiotensin system is activated in diabetes, and local angiotensin II production may lead to oxidative damage *via *the angiotensin II type 1 receptor. Basic and clinical data indicate that angiotensin II receptor blockers have the potential to preserve left ventricular function and prevent cardiac remodeling that is exaggerated by oxidative stress in patients with diabetes. Thus, alleviation of oxidative stress might be one possible strategy in the treatment of diabetic patients associated with sleep apnea.

## INTRODUCTION

Diabetes mellitus is a leading cause of morbidity and mortality because of its vascular complications [1,2]. Although type 1 diabetes is an important clinical problem with numerous long-term complications [3], the vast majority of diabetic patients with vascular complications have type 2 diabetes [4,5]. Diabetes mellitus currently affects 171 million persons worldwide, and there are predicted to be 366 million diabetic patients by the year 2030. In fact, the prevalence of type 2 diabetes is projected to double, especially in developing countries [6].

The most common cause of death among patients with diabetes is atherosclerotic cardiovascular disease. Current theories suggest that the initial event in atherogenesis is endothelial cell dysfunction, which can be induced by various insults, including diabetes, hyperlipidemia, hypertension, and smoking [7-10]. It has been suggested that hyperglycemia, hyperinsulinemia and insulin resistance, glycation of proteins, oxidative stress, inflammation, and many other factors may be related to atherogenesis in diabetes [11].

Recently, obstructive sleep apnea syndrome (OSAS), which is often found in obese people, has been identified as an independent risk factor for cardiovascular disease [12,13]. We have reported that intermittent hypoxia increases oxidative stress and induces left ventricular remodeling in an experimental model of sleep apnea [14]. The present review focuses on the role of oxidative stress in diabetes mellitus and its regulation from the viewpoint of cardioprotection.

## OXIDATIVE STRESS AND CARDIOVASCULAR DISEASE IN DIABETES

### Increased Oxidative Stress in Diabetics

Hyperglycemia seems to promote an imbalance between the generation and elimination of reactive oxygen species (ROS). Oxidative stress in diabetes could arise from a variety of mechanisms, such as excessive production of ROS from the auto-oxidation of glucose, glycation of proteins, and glycation of antioxidant enzymes (limiting their capacity to detoxify ROS). These changes could result in damage to cellular organelles and membranes, which may lead to diabetic complications [15].

Hyperglycemia is a key clinical manifestation of diabetes mellitus, and it stimulates several pathways. The polyol pathway is one of the pathways by which ROS increase in hyperglycemia: it involves conversion of glucose to sorbitol by aldose reductase and consumes NADPH, which acts as a coenzyme in the production of reduced glutathione, with the resulting depletion of NADPH causing an increase of oxidative stress through inadequate catalysis of H_2_O_2_ [16]. Also, increased conversion of sorbitol to fructose by sorbitol dehydrogenase leads to an increase of diacylglycerol (DAG), and activates protein kinase C (PKC), which might in turn induce the activation of NADPH oxidase and increase oxidative stress by decreasing the NAD+/NADH ratio [17].

Increased production of fructose, the end production of the polyol pathway, leads to an increase of advanced glycation end-products (AGEs), and ROS might also be produced during the creation of AGEs [18]. AGEs could generate ROS directly or *via* the receptors for AGEs (RAGE) [19,20]. In addition, AGEs promote the migration, proliferation, and differentiation of smooth muscle cells, the production of several cytokines, induction of adhesion molecule expression, and production of extracellular matrix (ECM) through RAGE or the scavenger receptor.

Recently, it has been shown that vascular smooth muscle cells and endothelial cells can produce ROS through activation of NADPH oxidase, which seems to be the most important source of ROS in intact arteries rather than enzymes involved with arachidonic acid (xanthine oxidase) or release from mitochondrial sources [21,22]. Inoguchi *et al*. [23] have shown that a high glucose level stimulates ROS production through activation of PKC-dependent NADPH oxidase in both vascular smooth muscle cells and endothelial cells, and they have also shown that the increase of ROS production by high glucose is completely reversed by diphenylene iodonium, an NADPH oxidase inhibitor. The increase of free radical production by exposure to high glucose was also completely blocked by a specific PKC inhibitor, suggesting that there was PKC-dependent activation of NADPH oxidase. On the other hand, Nishikawa *et al*. [24] reported that normalizing mitochondrial superoxide production blocks glucose-induced activation of PKC, increases the formation of AGEs, and promotes the polyol pathway. Thus, oxidative stress is increased in patients with diabetes mellitus through many pathways, and which of these should become the therapeutic target remains controversial.

### Vascular Damage by Oxidative Stress

Oxidative stress in diabetics induces thrombogenesis, endothelial dysfunction, and vascular inflammation [25]. Nitric oxide (NO) has an important role in protecting the vasculature against atherosclerosis, and endothelial NO synthase (eNOS) is responsible for most vascular NO production. Superoxide reacts with vascular NO to form peroxynitrite, and the cofactor tetrahydro-L-biopterin (BH4) is highly sensitive to oxidation by peroxynitrite. A decrease of the BH4 level promotes superoxide production by eNOS [26]. Both mechanisms lead to the loss of NO bioactivity, which might induce endothelial dysfunction and atherosclerosis [27].

ROS are reported to induce the expression of various growth-related genes, including c-fos, c-myc, and c-jun [28-30]. Furthermore, ROS production *via* NADPH oxidase has been implicated in the pathogenesis of angiotensin II-induced hypertension and vascular smooth muscle hypertrophy.

In endothelial cells, cytokine-induced expression of vascular cell adhesion molecule-1 (VCAM-1) has been reported to involve mobilization of nuclear factor-kappa B (NF-κB) through ROS and can be blocked by an antioxidant. Expression of VCAM-1 promotes the adhesion of monocytes to endothelial cells and may be important in the development of atherosclerosis. These findings suggest that an increase of ROS production *via* NADPH oxidase in vascular cells may contribute to the acceleration atherosclerosis in patients with diabetes.

### Myocardial Damage by Oxidative Stress

Oxidative stress related to hyperglycemia has been implicated as a major factor in the pathogenesis of cardiac hypertrophy and diabetic cardiomyopathy [15], which is not accompanied by either hypertension or coronary artery disease [31]. Diabetes is a well-known risk factor for the development of heart failure. Indeed, the Framingham Heart Study showed that the frequency of heart failure is twice as high in diabetic men and five times as high in diabetic women compared with age-matched control subjects [32]. Gonzalez-Vlilchez *et al*. [33] reported that diabetics developed concentric left ventricular hypertrophy and with impaired systolic and diastolic function. Diabetic cardiomyopathy is a major reason for the high morbidity and mortality of diabetics. Factors that are involved in the development of diabetic cardiomyopathy include impaired calcium homeostasis, upregulation of the renin-angiotensin system (RAS), increased oxidative stress, altered substrate metabolism, and mitochondrial dysfunction [31]. Several groups have shown that overproduction of ROS occurs in both type 1 and type 2 diabetes [31].

Recently, it was suggested that myocardial dysfunction may play an important role in the pathogenesis of impaired cardiac contractility in diabetics [34]. Boudina *et al*. [35] reported that decreased mitochondrial respiration and reduced expression of proteins involved in oxidative phosphorylation were observed in obese type 2 diabetic mice, and stated that such changes might contribute to cardiac dysfunction *via* reduced ATP production. Under physiological conditions, most of the ROS generated within a cell come from the mitochondria. Increased mitochondrial generation of ROS has been demonstrated in various tissues exposed to hyperglycemia [36]. Nitration of mitochondrial proteins (an index of oxidative damage) is increased in the hearts of diabetic mice [37]. Because mitochondrial hydrogen peroxide production is increased and glutathione levels are reduced in diabetic hearts, the source of ROS has been suggested to be the mitochondria [38]. Non-mitochondrial sources of ROS, including increased AGE formation, increased PKC isoform expression, and increased hexosamine pathway flux, have also been suggested to play a role in the diabetic heart [39]. Increased ROS generation activates maladaptive signaling pathways, which might lead to cell death and thus contribute to the development of diabetic cardiomyopathy.

Increased ROS generation activates maladaptive signaling pathways, which might lead to cell death and thus contribute to the development of diabetic cardiomyopathy. An increase of apoptosis, an increase of DNA damage, and reduced activity of the DNA repair pathway have been reported in diabetic animals [40]. ROS activate NF-κB, which plays a crucial role in mediating the immune and inflammatory responses, as well as apoptosis. The c-jun NH(2)-terminal kinases (JNK) and p38 MAPKs, which are members of the complex superfamily of MAP serine/threonine protein kinases, are stimulated by ROS. The pathways mediated by NF-κB, JNK, and p38 MAPK are potential stress-signaling systems that could have a role in the late complications of diabetes [39].

## SLEEP APNEA SYNDROME AND DIABETES 

**Fig. (1):**
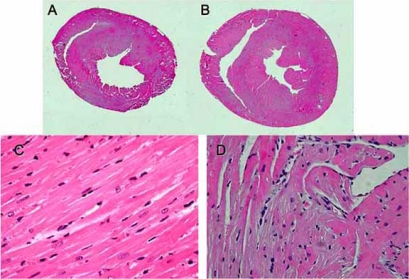
Representative macrographs (A, B) and light micrographs (C, D) of hearts from the diabetic rats. The diabetic rats kept under normoxia exhibited nearly normal morphology (A, C). Hypoxia caused cardiac hypertrophy, disarrangement of myofibers, and increased interstitial fibrosis (B, D). original magnification; x 100.

**Fig. (2):**
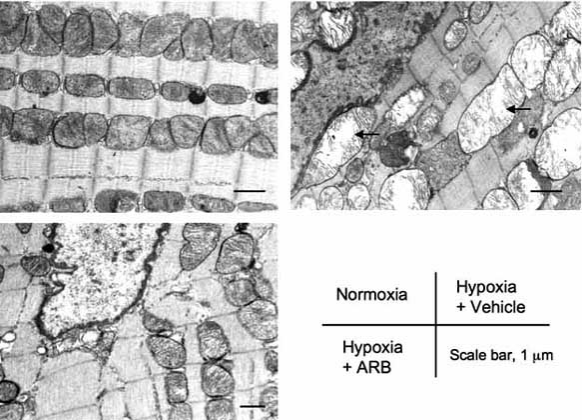
Electron micrographs of the left ventricular (LV) myocardium in diabetic rats. In diabetic rats kept under normoxia, mild deformity of mitochondria was observed. Hypoxia induced ballooning and loss of cristae in many mitochondria (arrows). Treatment with angiotensin-II receptor blocker (ARB) preserved the fine structure of the LV myocardium. Reproduced from Inamoto S, Hayashi T, Tazawa N, *et al.* Angiotensin- II receptor blocker exerts cardioprotection in diabetic rats exposed to hypoxia. Circ J 2006; 70: 787-792.

Obstructive sleep apnea syndrome (OSAS) is characterized by recurrent episodes of upper airway obstruction during sleep that induce hypoxia. Coughlin *et al*. [41] reported that OSAS was closely associated with an increased prevalence of metabolic syndrome. Metabolic syndrome is a cluster of risk factors for atherosclerotic cardiovascular disease, and this syndrome contributes to the development of diabetes mellitus [2]. In addition, we previously reported that continuous exposure to hypoxia causes the acceleration of myocardial degeneration in diabetic rats [42] (Figs. **1** and **2**). These findings suggest that a strong relationship may exist between OSAS and diabetes.

OSAS patients have significantly higher fasting blood glucose and insulin levels compared with obese controls [43]. Polotsky *et al*. [44] have shown that intermittent hypoxia due to OSAS exacerbates insulin resistance and glucose intolerance associated with obesity in the presence of leptin deficiency. On the other hand, several studies have demonstrated that OSAS is associated with insulin resistance and glucose intolerance independently of obesity [45-47]. Intermittent hypoxia due to OSAS rather than obesity might play an important role in the development of diabetes by inducing insulin resistance and glucose intolerance. Patients with OSAS have elevated plasma levels of TNF-α and IL-6 [48,49], and such inflammatory cytokines may be responsible for the development of diabetes. In particular, TNF-α has been reported to inhibit insulin signaling [50,51]. Therefore, TNF-α is likely to be crucial for the pathogenesis of diabetes in patients with OSAS. Further studies are needed to better clarify the role of inflammatory cytokines in both OSAS and diabetes.

## HYPOXIA AND REACTIVE OXYGEN SPECIES 

The hyperglycemic state contributes to cardiovascular complications in patients with OSAS. Intermittent hypoxia due to OSAS is known to be an independent risk factor for cardiovascular disease, including hypertension, congestive heart failure, and stroke [52]. We previously reported that hypoxia induced LV remodeling in diabetic rats and atherogenic mice [42, 53]. In addition, hypoxia accelerates the progression of atherosclerosis in atherogenic mice [54].

Overproduction of ROS causes oxidative stress, and has been implicated in the pathophysiology of cardiovascular disease. Recent studies have revealed that intermittent hypoxia increases ROS production [55], lipid peroxidation, and isoprostane levels [56] in the brains of experimental animals. In addition, Chen *et al*. [57] demonstrated an increase of oxidative stress in the hearts of rats exposed to intermittent hypoxia. These results suggest that oxidative stress may play a crucial role in the development of cardiovascular disease among patients with OSAS.

**Fig. (3):**
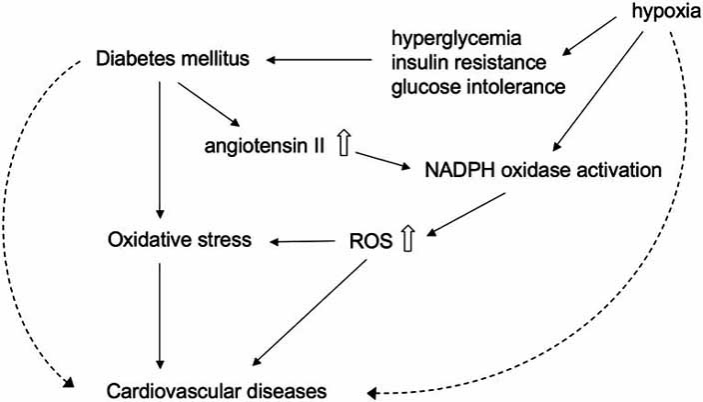
Role of oxidative stress in the progression of cardiovascular diseases accompanied by diabetes mellitus. Diabetes mellitus induced oxidative stress at least partly through NADPH oxidase activation, and consequently accelerated the progression of cardiovascular diseases. In addition, hypoxia might be implicated in the development of diabetes mellitus and production of reactive oxygen species (ROS), associated with the insult to mitochondria in cardiomyocytes by hypoxia itself.

NADPH oxidase is a major producer of ROS. This enzyme is composed of two membrane-bound subunits (gp91 phox and p22phox), as well as four cytosolic subunits (p40phox, p47phox, p67phox, and rac-1). Both angiotensin II and inflammatory cytokines have already been shown to stimulate NADPH oxidase, while hypoxic stress may be similarly important for its activation. In fact, Zhan *et al*. [58] reported that NADPH oxidase-derived ROS contribute to oxidative injury in the brains of mice exposed to intermittent hypoxia. Moreover, we have shown that hypoxia increases ROS production by NADPH oxidase in the aorta and LV myocardium, and consequently accelerates both atherosclerosis and LV remodeling [53,54]. Thus, intermittent hypoxia might enhance oxidative stress at least partly through activation of NADPH oxidase. Oxidative stress is also responsible for the activation of NF-κB [59,60], which regulates the expression of inflammatory cytokines and mediates monocyte-endothelial cell adhesion. We observed that hypoxia activates NF-κB in the LV myocardium of atherogenic mice [53]. This raises the possibility that NF-κB is an essential factor for the development of cardiovascular disease associated with hypoxic states. Taken together, these findings suggest that cardiovascular disease might be promoted by oxidative stress related to intermittent hypoxia (Fig. **3**). In addition, NADPH oxidase might be a useful target for therapeutic intervention to prevent cardiovascular disease in patients with OSAS.

## NOVEL THERAPEUTIC STRATEGIES FOR OXIDATIVE STRESS 

Oxidative stress has now been proved to play an important role in the development and progression of myocardial remodeling in patients with diabetes [61-63] (Fig. **3**). There is a growing body of evidence suggesting that antioxidants exert a protective effect in experimental model of heart failure [63-65]. Studies on myocardial ischemia-reperfusion injury have demonstrated the potential therapeutic value of radical scavengers, antioxidant extracts from a variety of plants, and polyphenols from food and wine, as well as vitamin E, vitamin C, and beta-carotene [66-69].

Haidara *et al*. [63] reviewed this area and concluded that administration of antioxidants might have a cardioprotective effect in the experimental setting and might protect against endothelial dysfunction associated with atherosclerosis, thus providing an effective means of reducing cardiovascular complications in diabetics.

In the clinical trials performed so far, however, the efficacy of treatment with antioxidants has been variable, probably due to inadequate doses and incorrect protocols, so these agents might still be promising [70-73]. Achieving the same beneficial outcome in the clinical setting might require a different approach that targets more specific intracellular pathways, in addition to the scavenging of excess oxygen radicals.

It has been shown that intensified multifactorial intervention with tight glucose regulation, renin-angiotensin system blockers, aspirin, and lipid-lowering agents can reduce the risk of nonfatal cardiovascular disease in patients with type 2 diabetes [74-76]. Recently, Gæde *et al*. [77] reported that intensive intervention with multiple drugs and behavior modification had a sustained beneficial effect on vascular complications, as well as reducing the all cause death rate and the cardiovascular death rate. Thus, it is obvious that more research is required to evaluate the efficacy of antioxidants in patients with diabetes.

### Angiotensin-converting Enzyme (ACE) Inhibitors and Angiotensin-II Receptor Blockers (ARB)

Activation of RAS and the subsequent increase of angiotensin II and aldosterone levels contribute to changes of the insulin/IGF-1 signaling pathway and promote the formation of ROS that induce endothelial dysfunction and cardiovascular disease [78]. Angiotensin-II is known to increase the expression of adhesion molecules, cytokines, and chemokines and it exerts a proinflammatory effect on leucocytes, endothelial cells, and vascular smooth muscle cells. The inflammatory cascade is initiated by angiotensin-II *via* its type 1 receptor, followed by increased production of ROS and activation of NF-κB, which mediates the transcription and expression of various genes [79].

**Fig. (4):**
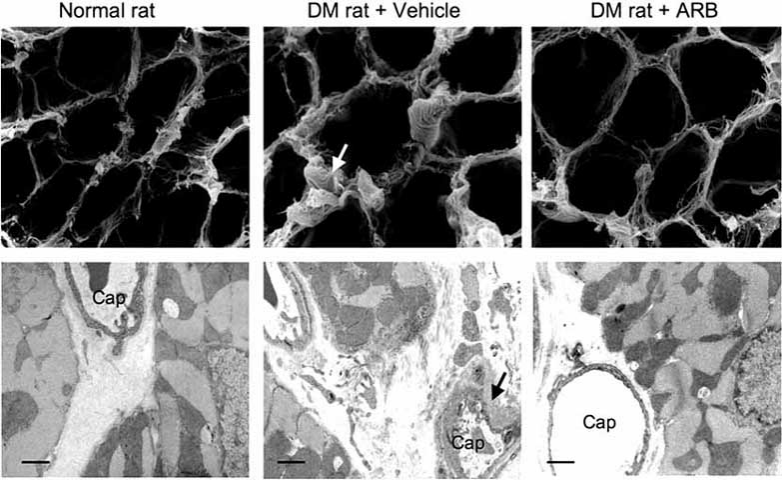
Representative scanning (above) and transmission (below) electron micrographs. Compared with normal rats, increased interstitial fibrosis (white arrow) and thickened basement membrane (black arrow) of capillary (Cap) were observed in diabetic (DM) rats. Treatment with angiotensin-II receptor blocker (ARB) suppressed the interstitial fibrosis and preserved the capillary basement membrane thickness. Scale bar=1 µm. Reproduced from Hayashi T, Sohmiya K, Ukimura A, *et al.* Angiotensin II receptor blockade prevents microangiopathy and preserves diastolic function in the diabetic rat heart. HEART 2003; 89: 1236-42.

RAS activation is important for the progression of cardiovascular pathology along the continuum from the existence of hypertension and other risk factors to end-stage cardiovascular disease [80]. Many studies have shown that blockade of angiotensin-II significantly reduces the levels of proinflammatory mediators and oxidative stress products in various models of inflammation. We previously reported that administration of the ARB candesartan intraperitoneally *via* an osmotic minipump prevented microangiopathy and preserved diastolic function in diabetic rats [81]. Candesartan was also effective for improving cardiomyocyte diameter and decreasing the levels of inflammatory cytokines, such as IL-1β and IL-6. Transmission and scanning electron microscopy clearly showed the cardioprotective effect of ARB therapy (Fig. **4**).

Recently, we reported that ARBs could reduce oxidative stress and ameliorate hypoxia-induced left ventricular remodeling, partly through the inhibition of NF-κB and matrix metalloproteinase (MMP)-9, in diabetic rats and atherogenic mice [42,53]. Thus, ARBs might provide effective cardioprotection even under hypoxic conditions, such as in diabetic patients with sleep apnea.

In clinical trials, blocking angiotensin-II by treatment with ACE inhibitors or ARBs has been found to be beneficial for patients with various inflammatory diseases [82]. RAS blockade delays or avoids the onset of type 2 diabetes, and also prevents cardiovascular or renal events in diabetic patients [83-85]. Furthermore, recent studies have shown that ARB therapy reduces the frequency of atrial fibrillation and stroke [86-88]. Thus, inhibition of the RAS by administration of ACE inhibitors or ARBs represents first-line treatment for hypertensive target organ damage and progressive cardiovascular disease [89].

### Statin Therapy

Statins reduce the plasma cholesterol level by inhibiting 3-hydroxy-3-methylglutaryl coenzyme A (HMG-CoA) reductase. Besides lowering cholesterol, statins are known to modify endothelial function and atherogenesis, stabilize atherosclerotic plaques, and reduce inflammation and thrombosis [90,91]. Recent studies have shown that intermediate products of the mevalonate pathway cause the activation of Rac1, a subunit of NADPH oxidase, leading to the production of ROS [92]. Accordingly, statins reduce oxidative stress by inhibiting NADPH oxidase.

In animal models, statins have been shown to ameliorate oxidative stress, prevent the progression of cardiac hypertrophy, and improve left ventricular function, which are all actions that might be beneficial in patients with heart failure [93]. Horiuchi *et al*. [94] reported that a combination of low-dose ARB and low-dose statin therapy acted synergistically to block neointimal growth. These “pleiotropic” properties of statins may have important clinical implications in addition to their use for lowering cholesterol levels.

Several clinical studies have demonstrated an increase of AT1 receptor expression in hypercholesterolemic men [95]. Subgroup analysis of the Heart Outcomes Prevention Evaluation (HOPE) study indicated that a beneficial effect of ACE inhibition was more evident in patients with concominant statin therapy [96]. Nickenig [91] has suggested that a combination of ARB and statin could be beneficial for patients with type 2 diabetes. Thus, further studies are warranted to confirm the beneficial impact of ARBs and statins through potentially synergistic modes of action, since these drugs could be used for potent and effective combination therapy in a variety of patient populations.

### Other Agents (Acarbose, Edaravone, and Calcium Channel Blockers)

Hyperglycemia may induce the generation of free radicals such as superoxide (O_2_^-^) and the hydroxyl radical. The interaction of glycated proteins with their cell surface binding sites may also lead to oxidative stress. Treatment with an α-glucosidase inhibitor might not only be useful for preventing postprandial hyperglycemia but also for suppressing oxidative stress in diabetics. Large-scale clinical trials have shown an impressive vasculoprotective effect of acarbose [97,98]. Recently, Rösen *et al*. [99] reported that treatment of insulin-resistant rats with acarbose prevented an excessive rise of the plasma glucose level and the initiation of a reaction starting with the generation of ROS and spreading to affect the whole cell.

Edaravone (3-methyl-1-phenyl-2-pyrazolin-5-one) is a free radical scavenger that traps hydroxyl radicals, as indicated by its inhibition of the formation of hydroxylated salicylate. Edaravone has been reported to exert a protective effect against cerebral and myocardial ischemia in rats. We have also reported that edaravone effectively decreased the activity of inducible nitric oxide synthase in the left ventricular myocardium of type 2 diabetic rats and preserved the ultrastructure of the mitochondria [100]. Thus, edaravone has a modest cardioprotective effect on the hearts of diabetic animals.

Both clinical studies and basic research have revealed that calcium antagonists not only protect the endothelium through their hypotensive action, but also improve endothelial function through stimulation of NO production [101]. Although the precise vasoprotective mechanisms of calcium channel blockers are still obscure, recent studies have suggested that nifedipine might stimulate SOD expression in endothelial cells *via* enhancement of VEGF expression by vascular smooth muscle cells, and thus might reduce oxidative stress and increase NO production [102].

Therefore, α -glucosidase inhibitors, free radical scavengers, and calcium channel blockers should also be included as candidates for antioxidant therapy in the treatment of patients with diabetes.

## CONCLUSION

Of course, meticulous glycemic control is important to prevent cardiovascular remodeling in diabetes. As reviewed above, oxidative stress has also been proved to play an important role in the development and progression of cardiovascular remodeling. New insights into the mechanisms that increase oxidative stress in diabetes might lead to novel treatment strategies. In conclusion, alleviation of oxidative stress should be taken into consideration as one of the possible strategies for the treatment of patients with diabetes, especially when they are exposed to intermittent hypoxia due to the presence of sleep apnea.
